# Les accidents aux AVK: étude rétrospective à propos de 30 cas

**Published:** 2012-02-15

**Authors:** Issam Serghini, Younés Aissaoui, Youssef Quamouss, Rachid Sedikki, Nourredine Taj, Jaafar Salim Alaoui, Mohamed Zoubir, Mohamed Boughanem

**Affiliations:** 1 Service d’Anesthésie réanimation, hôpital militaire Avicenne, Marrakech, Maroc

**Keywords:** Anti-vitamine K, hémorragie, surveillance, Maroc

## Abstract

Les accidents hémorragiques sous AVK (antivitamines K) sont la première cause d’hospitalisation iatrogène. Le but de cette étude est de ressortir les caractéristiques des patients présentant des accidents hémorragiques graves sous AVK et de mettre le point sur la prise en charge. Nous avons réalisé une étude rétrospective portant sur 30 malades, réalisée au service de réanimation de l’hôpital militaire Avicenne de Marrakech. L’âge moyen de nos patients est de 59, 6 avec un sexe ratio de 1,5 (18 femmes et 12 femmes). On note dans notre série le terrain multi-taré des patients sous AVK avec plusieurs antécédents en cause. La fibrillation auriculaire était la principale indication recensée. Plus que la moitié ne bénéficiaient pas d’une bonne surveillance biologique à base d’INR (international normalized ratio). Le saignement d’origine gastro-intestinal était le plus fréquent. La conduite était différente en fonction des cas mais axée sur l’arrêt des AVK, l’administration de vit K, la transfusion de culots globulaires et plasma frais congelé. Plus du quart des patients admis sont décédés. L’index de Landefeld s’est avéré utile, il permet de classer les patients selon le risque prédictif de saignement (élevé moyen ou faible), ce qui concordait avec les résultats de notre étude. Son importance est d’ autant plus qu’il est facile à mesurer et à appliquer par le médecin en ambulatoire et permet de définir les patients nécessitant une surveillance accrue. La polymédication et les antécédents de saignement digestif sont apparus comme facteur de risque de saignement sous AVK. La prévention de la survenue de ces accidents est le pilier de la prise en charge, d’où l’importance de l’information et de l’éducation des différents intervenants dans cette complication iatrogène potentiellement mortelle.

## Introduction

Les antivitamines K (AVK) sont des anticoagulants administrés par voie orale, utilisés pour la prévention de la thrombose veineuse ou de son extension [1]. Une augmentation importante de leur utilisation a été notée au cours des deux dernières décennies, résultat du vieillissement de la population et de l’élargissement de leurs indications. Des progrès ont été réalisés pour sécuriser cette utilisation, comme l’identification de la meilleure cible thérapeutique, la définition des indications à travers des études randomisées et l’établissement de modèles de gestion d’anticoagulation. Malgré cela, le traitement par AVK présente encore un lourd pourcentage d’accidents dont les plus graves sont les accidents hémorragiques [2,3].

## Méthodes

Le but de notre étude est de ressortir les caractéristiques des patients présentant des accidents hémorragiques sous AVK, faire le point sur la prise en charge de cet accident grave, ressortir les facteurs pronostics des patients présentant des complications hémorragiques. Il s’agit d’une étude rétrospective portant sur 30 patients au service de réanimation de l’hôpital militaire Avicenne de Marrakech. Les patients inclus étaient ceux mis sous AVK et qui ont présentés une complication aux AVK de type hémorragique. Etaient exclus de notre étude les accidents hémorragiques aux héparines seules ou associés aux anti-vitamines K et les patients avec un INR au-delà de la marge thérapeutique sans manifestations hémorragiques (surdosage asymptomatique). Pour chaque patient une fiche de recueil des données a été utilisée. Elle comprenait les données anthropométriques, les antécédents, l’indication et la dose de l’AVK, l’ancienneté de sa prescription et les traitements associés. Les paramètres biologiques recensés étaient principalement l’INR (avant et au moment de l’accident), le temps de Quick, le taux d’hémoglobine et le taux de plaquettes.la fiche comportait également des données sur la complication hémorragique (gravité et localisation). Ont été colligés également la conduite thérapeutique (l’arrêt ou pas de l’AVK), les doses reçues de vitamine K, le nombre de culots globulaires et celui des unités de plasmas frais congelé ainsi que l’évolution des patients.

## Résultats

Trente patients ont été inclus dans cette étude, tous hospitalisés durant la période s’étendant entre le 27/05/2006 et le 10/04/2010.Sur l’ensemble de ces patients, On note 60 % de femmes et 40 % (18 femmes et 12 hommes), soit un sexe ratio de 1,5. La moyenne d’âge était de 59, 6 ans, avec des âges extrêmes allant de 31 à 75 ans. Leurs antécédents étaient variés (Tableau 1).

**Tableau 1:** Les antécédents des patients

Antécédents	Nombre	Pourcentage
Hypertension artérielle	14	46 ,66
Insuffisance cardiaque	8	26 ,66
Diabète	8	26 ,66
Tabac	7	23 ,33
Chirurgie cardiaque	7	23 ,33
Alcool	5	16 ,66
Insuffisance rénale	4	13 ,33
Accident vasculaire cérébral	3	10
Hémorragie digestive	9	30

**Figure 1:**
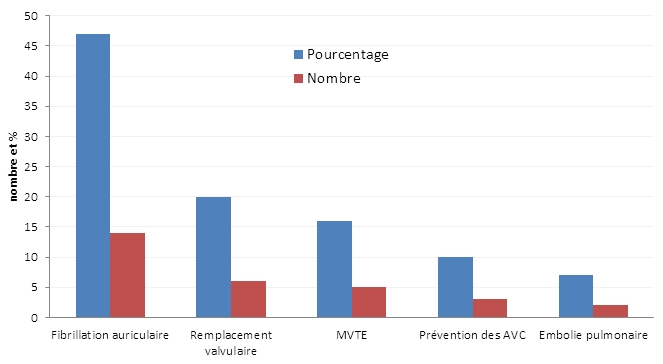
Indications des antivitamines K

La dose moyenne de L’AVK était de 0,891 comprimé par jour. Le rythme d’administration était soit alterné (20%), double (11%) ou unique (69%). La durée moyenne du traitement était de 30,23 mois avec des extrêmes allant de 20 jours à 11 ans. Les indications des AVK étaient essentiellement la pathologie cardio-vasculaire dans 66,66 % des cas (Figure 1). L’utilisation de l’index de Landefeld a montré qu’on pouvait définir un risque prédictif de saignement. Nous avons appliqué cet index sur notre série de patients (Tableau 2).

**Tableau 2:** Index de Landefeld

	Age 65 ans	ATCD AVC	ATCD saignement	Co-morbidité	Index de Landefeld
Cas 1	−	+	+	+	3
Cas 2	+	+	+	+	4
Cas 3	+	+	−	+	3
Cas 4	−	−	−	+	1
Cas 5	−	−	−	+	1
Cas 6	+	+	−	+	3
Cas 7	−	−	+	+	2
Cas 8	−	+	+	+	3
Cas 9	+	+	+	+	4
Cas 10	−	−	+	−	1
Cas 11	−	−	+	+	2
Cas 12	−	−	−	+	1
Cas 13	−	−	−	−	0
Cas 14	+	−	+	+	3
Cas 15	−	−	+	+	3
Cas 16	−	−	−	−	0
Cas 17	+	−	+	+	3
Cas 18	−	−	+	+	2
Cas 19	−	−	−	+	1
Cas 20	−	−	+	+	2
Cas 21	+	+	+	−	3
Cas 22	+	−	+	+	3
Cas 23	+	+	+	+	4
Cas 24	+	−	+	+	3
Cas 25	−	−	−	−	0
Cas 26	−	−	−	−	0
Cas 27	−	+	−	−	1
Cas 28	−	−	−	−	0
Cas 29	+	−	+	+	3
Cas 30	−	−	+	+	2

ATCD : Antécédents, AVC: Accident vasculaire cérébrale.

**Figure 2:**
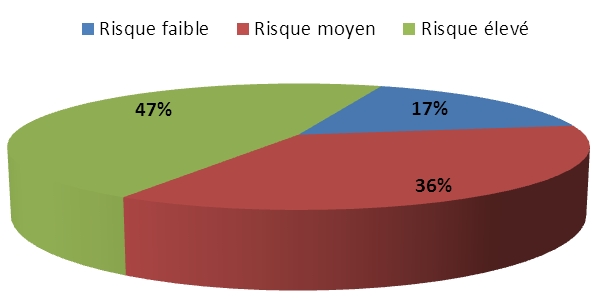
Risque de saignement secondaire selon l’index de Landefeld

**Figure 3:**
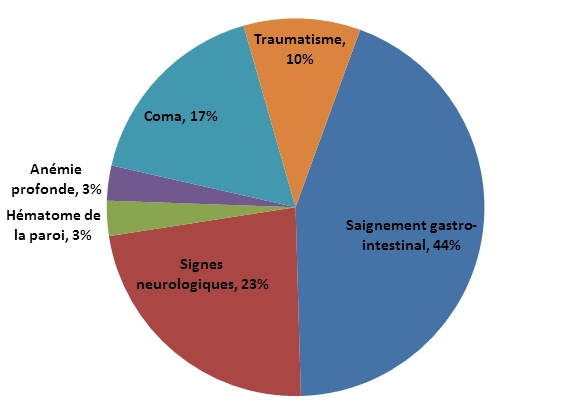
Motifs d’admission aux urgences

Le risque de saignement était évalué selon cet index (Figure 2). 76,6% des patients étaient polymédicamentés (Trois familles médicamenteuses ou plus) au moment de l’accident. 13,33 % prenaient un ou deux médicaments et 10% n’en prenaient aucun (Tableau 3). Le saignement par le tractus gastro intestinal était le plus fréquent (44%) mais d’autres motifs d’admission figuraient : Les signes neurologiques (23 %) ; le Coma (17%) ; le Traumatismes (10%); l’anémie profonde (3 %) et l’hématome de la paroi (3 %) (Figure 3). Dans 97%, l’accident correspondait à une manifestation hémorragique. L ‘hémorragie était extériorisée dans 54 % des cas et interne dans les 43 % restant. 60 % des patients n’avaient pas une mesure INR récente et ne bénéficiaient pas d’un suivi régulier. Sur les INR recensés précédant la survenue de l’accident, la moyenne était de 2,27 (extrême : 1,4 – 3 1,5). La moyenne de l’INR à l’admission des patients était de 4,98 (extrême: 3 – 12,39).

**Tableau 3:** Prise médicamenteuse concomitante

Médicaments	Fréquence	Pourcentage
Aucun	3	10
Antidiabétique oral	3	10
Insuline	5	16.66
Aspirine	13	43.33
Bétabloquant	10	33.33
Diurétique	17	56.66
Ciprofloxacine	8	26.66
Dérivés nitrés	11	36.66
Inhibiteurs de l’Enzyme de Conversion	6	20
Digitaliques	9	30
Cordarone	4	13.33
Ranitidine	3	10
Oracilline	7	23.33

Le TP à l’admission était en moyenne à 32 ,85 %. Le taux d’hémoglobine était de 7,31g/dl. Pour tous les malades, le traitement anticoagulant a été arrêté. Près d’un quart des patients (26,66 %) ont été transfusés par des culots globulaires et 12 soit 40 % ont reçu des unités de plasma frais congelé. Seulement 10 patients (33, 33 %) ont reçu de la vitamine K. Deux patients l’ont reçu par voie orale et les huit autres par voie intraveineuse. Le traitement anticoagulant par l’héparine fractionnée a été repris au bout de 29 heures en moyenne chez 28 patients. Le chevauchement avec l’acénocoumarol a été entrepris chez 19 patients en moyenne 96 heures après l’accident. Près d’un quart des patients (26,66%) ont dû bénéficier d’une chirurgie d’hémostase. 12 patients sont décédés au cours de leurs hospitalisations, avec onze décès directement imputables aux AVK, 1 décès survenu après stabilisation de l’accident hémorragique, et qui est lié à une infection nosocomiale contractée lors de son séjour au service de réanimation.

## Discussion

En France, les accidents hémorragiques imputables aux AVK représentent la première cause d’accident iatrogène. Ils sont responsables de 20000 hospitalisations/an, avec une incidence d’épisodes hémorragiques graves de 5/100 patients/an et d’accidents mortels de 1/ 100 patients/an [4]. L’Italian Study on Complications of Oral Anticoagulant Therapy a montré que le risque de décès, de complications majeures et de complications mineures en rapport avec les AVK était respectivement de 0,25; 1,1 (0,1% par mois de traitement) et 6,2 (0,5% par mois de traitement) pour 100 patients année [5]. La diminution de la prévalence de l’hémorragie va avec une meilleure connaissance des facteurs de risque hémorragique chez un patient sous AVK, d’une standardisation des objectifs et des moyens thérapeutiques.Le sexe du patient n’intervient pas dans la survenue des accidents des AVK. La nette prédominance féminine (60%) concorde avec d’autres études [6]; alors que les statistiques européennes sont tout à fait contraire: prédominance masculine pour Costecalde et coll [7] et Chaussade [8]. L’âge avancé est un facteur sensibilisant à l’action des AVK. Le risque d’accident hémorragique est trois fois plus fréquent dans la population de plus de 70 ans. Dans notre série, la moyenne d’âge était de 59,6 ans. L’âge est apparu comme facteur significatif de la survenue des accidents hémorragiques sous AVK, s’expliquant par le fait de l’existence d’une altération des fonctions cognitives chez le sujet âgé [9,10]. L’observance est diminuée en cas de polymédication, 76,66 % des patients étaient sous plus de 3 médicaments à la fois pour des pathologies diverses. La prescription des AVK doit tenir compte des contre-indications absolues et relatives qui tiennent au terrain et à l’état digestif hépatique est rénal. Berrutti, dans son étude portant sur 151 accidents hémorragiques dus aux anticoagulants a mis en évidence : une fragilité vasculaire dans 41 cas; Une HTA dans 27 cas; Un diabète et un éthylisme chronique dans 7 cas respectivement et 1 cas de maladie de Vaquez [11]. Dans notre étude, L’HTA a été retrouvée dans 46,66% et le diabète dans 26,66% ce qui confirme les donnée suscitées concernant la place des tares associées dans la survenue des accidents hémorragiques. Les résultats des différentes études cliniques ont permis d’établir des recommandations à propos de la prise en charge du traitement par les AVK. Les niveaux de preuves sur lesquels elles sont fondées et le degré de validité de ces recommandations ont été décrits par Cook et coll [12] (Tableau 4). Nos résultats concordent avec la littérature en ce qui concerne la principale indication des AVK qui est l’origine cardiaque [13].Dans notre série, 66,66% des indications sont d’origine cardiaque. Même si l’adoption de l’INR a remarquablement accru la fiabilité de la surveillance du traitement par les AVK, ce système connait des limites. Les concentrations élevées en citrate (3,8%) utilisées souvent en France mènent à des valeurs d’INR plus élevées, de la même manière qu’un tube de sang rempli excessivement.3,2 % de concentration de citrate et un tube rempli en juste proportions peuvent réduire cette difficulté.

**Tableau 4:** Indication des antivitamines K (AVK) et niveau d’hypocoagulation recommandé

Indication des AVK	INR* souhaité
Prévention primaire des thromboses veineuses (chirurgie à haut risque thrombotique)	2–3
Traitement des thromboses veineuses et des embolies pulmonaires	2–3
Prévention des embolies systémiques en cas de: Prothèse valvulaire tissulaireFibrillation auriculaire, Infarctus du myocarde, Cardiopathie valvulaire	2–3
Prothèse valvulaire mécanique (INR variable en fonction de la localisation de la valve et de la thrombogénécité de la prothèse)	2,5–4
Embolie systémique récidivante	3–4,5

INR: International Normalized ratio

Selon le consensus Nord-Américains, le premier contrôle par INR doit être réalisé après les 2 ou 3 prises initiales de l’anticoagulation [14]. Chez les patients hospitalisés, les contrôles seront réalisés tous les jours jusqu’a ce que l’INR soit dans la marge thérapeutique pendant au moins 2 jours successifs, puis 2 à 3 fois par semaine pendant une à deux semaines puis espacé jusqu’a un intervalle ne dépassant pas quatre semaine. Dans notre étude, 60% des patients n’avaient pas une mesure INR récente et ne bénéficiaient pas d’un suivi régulier avec une mauvaise observance, expliquant la survenue de ces accidents iatrogènes. L’hémorragie d’origine gastro-intestinale est la complication la plus fréquente notre étude avec 43,33%, ce qui correspond aux données de la littérature qui rapportent 60% de complications digestives. Ces hémorragies peuvent être extériorisées révélant une affection organique (ulcère 33% gastrite 2 % cancer 2% et hernie hiatale 10%) deux fois sur trois [15]. Sur les 13 patients qui avaient une localisation hémorragique digestive, 7 ont présenté une hématémèse sur ulcère ou sur gastrite. En deuxième position figure la complication neurologique avec 40% alors qu’elle ne représente que 12% dans l’étude de White et Coll. Les hémorragies intracérébrales répertoriées dans la littérature sont les hématomes sous duraux (les plus fréquent), les hémorragies sous-arachnoïdiennes et les hémorragies médullaires. Dans notre série, parmi les 12 patients qui ont présenté une hémorragie cérébrale, 7 avaient des HSD. Les hémorragies intracérébrales sont la cause la plus fréquente de décès et d’invalidité [16]. Parmi les 12 décès enregistrés de notre étude, 8 étaient dues à des complications neurologiques. Le seul patient qui a gardé des séquelles (porencéphaliques) est celui qui a présenté un saignement neurologique ce qui rejoint les données de la littérature.

La prolongation de la durée du traitement par les AVK augmente pour certains auteurs le risque hémorragique. Dans notre série, la fréquence des accidents hémorragiques s’est élevée à 46 % au-delà d’1 an de traitement. La majorité des auteurs suggèrent que c’est au début du traitement que l’incidence des hémorragies est la plus grande (le premier mois, voir les trois premiers mois) [17]. 23, 33% des hémorragies colligés dans notre étude, ont été observées durant les trois premiers mois. La classification de Landefeld permet de déterminer un risque de saignement majeur à 3 et 12 mois en additionnant les facteurs de risque suivant : âge supérieur à 65 ans, antécédents d‘AVC, antécédents de saignement gastro-intestinal, comorbidités (anémie insuffisance rénale ou hépatique, infarctus du myocarde). Chacun de ces items s’il est présent, est associé à un point. Elle permet de déterminer un risque faible (0 point); moyen (1 à 2 points) ou élevé (3 à 4 points) [18]. L’intérêt et la fiabilité de cet index s’est bien démontré à travers de notre étude, les patients ayant un risque prédictif de saignement élevé (3 à 4 points) ont présenté plus de complications hémorragiques majeurs par rapport aux patients ayant un risque prédictif moyen. La prise en charge de ces complications hémorragiques a fait l’objet de récentes recommandations en Avril 2008. La base du traitement médicamenteux est la Vit K parfois associées aux PPSB (Prothrombine Proconvertine Stuart B), et peut être complétée par un geste d’hémostase (artériographie avec embolisation, chirurgie d’hémostase).Les recommandations de l’HAS en avril 2008 portent sur trois situations majeurs : les surdosages asymptomatiques, la survenue d’une hémorragie, spontanée ou traumatique, associée ou non à un surdosage et la prise en charge lors d’une chirurgie ou d’un acte invasif [19]. La Vit K est les concentrés de complexe prothrombiques (CCP) ou prothrombine proconvertine Stuart B (PPSB) sont les moyens les plus appropriés plutôt que les PFC dans la mesure du possible (PFC n’est recommandé en France qu’en cas d’indisponibilité des PPSB). Les deux spécialités commercialisées en France sont le Kaskadil et l’Octapex. Il est recommandé d’arrêter l’AVK ; d’administrer en urgence un concentré de complexes prothrombiniques : 35 UI/Kg si l’INR initial est entre 2–3, 40 U/IKg s’il est entre 3–3,5, de 50 UI/Kg s’il est supérieur à 3,5 et d’administrer en urgence de la Vit K: 10 mg par voie veineuse ou 5 mg per os) [17].

Notre étude présente certaines limites, le choix de l’anticoagulant oral est un sujet d’actualité. IL prend en compte la demi-vie et la famille chimique. Les textes de consensus ont clairement indiqué les avantages des molécules à durée d’action longue ou intermédiaire en termes d’efficacité antithrombotique et de stabilité de l’effet anticoagulant. La warfarine est l’AVK de référence en raison de son efficacité. Au Maroc le seul AVK disponible est l’acénocoumarol (tous les patients étaient sous Sintrom). Ce paramétré n’a pu être testé. Le temps passé dans la zone thérapeutique est un indicateur précis du risque d’accidents hémorragiques ou thromboemboliques dans la plupart des études. Il peut être mesuré par plusieurs méthodes? la plus usuelle est le rapport entre la fraction des valeurs d’INR qui sont en dehors de la cible thérapeutique et la somme de tous les INR testés. C ‘est un excellent reflet de la qualité de la gestion de l’anticoagulation et permet d’identifier les patients à risque hémorragique ou thromboembolique. Nous n’avons pas pu l’évalué dans notre étude: la plupart des malades n’ayant pas respecté leurs contrôles biologiques.

## Conclusion

Les AVK sont utilisés depuis plus d’un demi-siècle dans la prévention des accidents thromboemboliques et sont l’une des thérapeutiques les plus efficaces. Cependant leur maniement est délicat et les risques de complications hémorragiques sont très présents. Nous avons essayé à travers cette étude de ressortir les caractéristiques des patients présentant des accidents hémorragiques sous AVK, de préciser les facteurs corrélés au risque hémorragique et enfin de faire le point sur la prise en charge de cet accident grave. Les nouveaux anticoagulants oraux offrent une autre alternative. Une bonne tolérance, une cinétique prévisible qui autoriseraient leur administration sans aucun contrôle biologique, l’absence de métabolisme hépatique, ajoutées à une efficacité équivalente en font des candidats potentiels pour remplacer les AVK dans les prochaines décennies.
